# Interleukin-10 at the crossroads of immunity in TLR7-induced lupus

**DOI:** 10.3389/fimmu.2026.1783769

**Published:** 2026-06-22

**Authors:** Johanna Pauline Williams, Anaïs Amend, Anna-Lena Schäfer, Paola Fernanda Ruiz-Aparicio, Antoine Nicolas Kraemer, Aileen Qian Luo, Laura Riechert, Lara Weber, Baerbel Keller, Rudolf Armin Manz, Reinhard Edmund Voll, Nina Chevalier

**Affiliations:** 1 Department of Rheumatology and Clinical Immunology, Medical Center - University of Freiburg, Faculty of Medicine, University of Freiburg, Freiburg, Germany; 2 Faculty of Biology, Albert-Ludwigs-University Freiburg, Freiburg, Germany; 3 Spemann Graduate School of Biology and Medicine (SGBM), University of Freiburg, Freiburg, Germany; 4 Center for Chronic Immunodeficiency, Medical Center - University of Freiburg, Faculty of Medicine, University of Freiburg, Freiburg, Germany; 5 Institute for Systemic Inflammation, University of Lübeck, Lübeck, Germany

**Keywords:** CD4 T cells, experimental lupus, interleukin-10 (IL-10), regulatory T cells, SLE, STAT, TLR7

## Abstract

**Objective:**

Systemic lupus erythematosus (SLE) is a prototypical autoimmune disease characterized by dysregulated innate and adaptive immunity. Soluble mediators like cytokines critically shape immune responses and sustain chronic inflammation. Interleukin-10 (IL-10), typically anti-inflammatory, also exerts pro-inflammatory effects in SLE, for example by promoting B cell differentiation and autoantibody production. Despite growing interest in IL-10 as a biomarker or therapeutic target, its dual roles have been reported inconsistently, likely reflecting differences in disease context, clinical presentation, disease activity, and the local microenvironment, an issue addressed in this work.

**Methods:**

To further clarify its role in lupus, we investigated its function in a toll-like receptor 7 (TLR7) agonist-induced murine model. By administering an antagonistic anti-IL-10 receptor (anti-IL-10R) antibody, we investigated its broad *in vivo* effects on the progression of lupus-like features and associated immune changes. We then examined the signaling pathways underlying observed immune alterations.

**Results:**

IL-10 showed both pro- and anti-inflammatory effects on immune cells; however, overall, it modestly limited disease progression, indicating that, under the given conditions, its regulatory, anti-inflammatory effects may slightly outweigh its pro-inflammatory actions. The most pronounced effects were seen in the CD4 T-cell compartment, where IL-10R blockade increased IL-17, while reducing regulatory T cell (T_reg_) frequencies, IL-10 and slightly also interferon gamma (IFN-γ) expression. Mechanistically, IL-10 activated similar downstream pathways in T effector cells (CD4^+^FoxP3^-^ T_eff_) and CD4^+^FoxP3^+^ T_reg_, with signal transducer and activator of transcription 3 (STAT3) signaling being most prominent. We speculate that the heightened sensitivity of T_regs_ to IL-10R blockade may stem from slightly elevated IL-10R expression.

**Conclusion:**

Collectively, these findings highlight dual role of IL-10 in lupus and suggest how a single cytokine may trigger opposing immune effects via shared signaling pathways. Further research is needed to clarify how a pro-inflammatory context directs IL-10 signaling toward stimulatory or suppressive outcomes, informing new therapeutic strategies for diseases such as SLE.

## 1 Introduction

Systemic lupus erythematosus (SLE) is a prototypical autoimmune disease with a complex and still not fully understood etiology. It results from the interplay of genetic and environmental factors that disrupt immune tolerance and trigger immune dysregulation. Key features include hyperactive autoreactive B and T cells and impaired innate responses, leading to the production of autoantibodies, especially anti-double-stranded DNA (anti-dsDNA). These autoantibodies form immune complexes (ICs) that promote systemic inflammation and cause multi-organ damage, affecting the kidneys, joints, blood, and central nervous system (1).

In addition to cellular immune components, soluble mediators such as cytokines play a central role in SLE pathogenesis by modulating innate and adaptive immune responses. This modulation drives chronic inflammation and disrupts the physiological balance essential for tissue homeostasis, thereby promoting disease progression (2–4). Key cytokines involved in SLE include pro-inflammatory mediators like type I interferons, interleukin 6 (IL-6), IL-17, and tumor necrosis factor alpha (TNF-α), as well as IL-10. Besides its anti-inflammatory properties, IL-10 exerts broad immunosuppressive effects by modulating antigen-presenting cells, inhibiting T cell activation and proliferation, thus reducing cytokine secretion and cytotoxicity and inducing long-term T-cell anergy via blockade of CD28 co-stimulatory signaling (5–7).

Accordingly, dysregulation or absence of IL-10 is critically implicated in the pathogenesis of various diseases, including chronic inflammatory, allergic, autoimmune, infectious, and neurodegenerative disorders, as well as malignant neoplasms (5, 8). The protective role of IL-10 has been well documented in chronic inflammatory bowel diseases (IBD) and is currently being explored as a therapeutic strategy in this context (9, 10). Similar beneficial effects have been reported in immune-mediated diseases such as asthma, psoriasis, and rheumatoid arthritis (8) as well as in experimental autoimmune encephalomyelitis (EAE) and neuritis (11).

However, emerging evidence points to a more complex, dual role for IL-10, balancing pro- and anti-inflammatory immune regulation. While IL-10 suppresses pro-inflammatory effector functions, it can also act as a pro-inflammatory immune modulator by promoting B cell differentiation and autoantibody production, particularly within extrafollicular responses, as well as enhancing cytotoxic T-cell responses and interferon gamma (IFN-γ) secretion (12–20). IL-10 can be synthesized by various immune cells, including CD4^+^ and CD8^+^ T cells, B cells, natural killer cells (NK cells), mast cells, as well as myeloid cells such as neutrophils, eosinophils, monocytes, macrophages, and dendritic cells. Monocytes, macrophages, and CD4^+^ T cells are often described as the primary sources of IL-10; however, this can vary depending on the inflammatory context (5, 8, 21, 22).

The cellular response to IL-10 (5, 8, 22, 23) is initiated by its binding to the IL-10 receptor (IL-10R), triggering downstream intracellular signaling cascades. The IL-10R is a heterodimeric cell surface receptor composed of two subunits: IL-10R1 and IL-10R2. IL-10R1 is primarily responsible for ligand recognition and is mainly expressed by lymphocytes, macrophages, and dendritic cells under basal conditions, though its expression can be upregulated upon activation. In contrast, IL-10R2 is constitutively expressed on nearly all cell types. IL-10 initially binds with high affinity to IL-10R1, triggering a conformational change that increases the complex’s affinity for IL-10R2. This sequential binding completes receptor assembly and initiates intracellular signal transduction (24, 25).

The IL-10 signaling pathway is primarily mediated through the activation of Janus kinases (JAKs) and STAT transcription factors (5, 8, 22). STAT3 primarily controls genes involved in cell survival, proliferation and inflammation (5, 26). Among its downstream targets is also suppressor of cytokine signaling 3 (SOCS-3), which inhibits mitogen-activated protein kinase (MAPK) activation, nuclear factor kappa-light-chain-enhancer of activated B cells (NF-κB) nuclear translocation, and pro-inflammatory gene expression. SOCS-3 also provides negative feedback by dampening STAT3 signaling. Beyond STAT3, IL-10R signaling can also activate STAT1 and STAT5, enabling formation of STAT heterocomplexes and diverse transcriptional responses (27). Additional IL-10-mediated pathways involve phosphoinositide 3-kinase (PI3K), protein kinase B (AKT), glycogen synthase kinase 3 (GSK3) and mechanistic target of rapamycin complex 1 (mTORC1) (28, 29).

Although IL-10 is increasingly recognized as both a biomarker and potential therapeutic target (30–32) in different clinical settings, current studies, including our own (21), report inconsistent findings, most likely reflecting its dual immunoregulatory and pro-inflammatory functions. These divergent effects likely depend on factors such as disease model, clinical presentation, activity level, microenvironment, and immune cell composition. To confirm and further delineate IL-10’s role, we investigated its impact on disease progression in an alternative murine lupus model and examined the underlying immune changes. In addition, we aimed to identify potential regulators of its stimulatory versus suppressive effects by analyzing the expression of downstream signaling pathways.

## 2 Materials and methods

### 2.1 Mice and models

Animal experiments were approved by the local governmental commission for animal protection of Freiburg (Regierungspräsidium Freiburg, approval no. G-22-108). All animals were bred in-house and housed under standard conditions with a 12-hour light/dark cycle in individually ventilated cages (IVCs), with food and water provided ad libitum. To induce TLR7-driven lupus, adult female C57BL/6J mice were topically treated with a TLR7 agonist (5% Imiquimod, ^®^Aksunim, 50 mg/g) for 12 weeks. The cream was applied to one ear three times per week at a dose of approximately 1.25 mg per application. To assess the role of IL-10, *in vivo* blockade of the IL-10R was performed during the final six weeks of Imiquimod treatment. Mice received weekly intraperitoneal injections of either anti–IL-10R antibody (150 µg/week; clone JES5-2A5, BioLegend) or a control substance (rat IgG, 150 µg/week, I4131/Sigma-Aldrich). In a separate cohort, anti–IL-10R or control substances were administered at a higher dose of 500 µg, but given only twice (six and three weeks prior to the endpoint). Mice were regularly (3x weekly) monitored for their health status, including body weight, fur condition, posture, hydration status, and behavioral signs. In addition, body weight and the development of proteinuria, as marker of disease/nephritis development, were recorded. Monitoring and treatment with imiquimod were performed on an alternating schedule by three investigators. Administration of the anti-IL-10R antibody was carried out in a blinded manner. Urine samples were collected by spontaneous urination. For a semiquantitative measurement of proteinuria, Albustix test strips (*Siemens*) were used. According to the color scale provided by the manufacturer, albuminuria was categorized as follows: 0–1 = trace, 1 = 30 mg/dl, 2 = 100 mg/dl, 3 = 300 mg/dl, and 4 > 2,000 mg/dl. Mice were deemed proteinuric after scoring a 2 on the color scale for at least two consecutive weeks. All mice of this study were euthanized at defined time points for organ collection and downstream analyses, none reached predefined humane endpoints that would have required earlier euthanasia. Mice were euthanized through CO_2_-inhalation (flow rate adjusted to replace between 30-70% of the chamber volume/min) or cervical dislocation in accordance with institutional and governmental guidelines. Humane endpoints included markedly increased or reduced locomotor activity, impaired social behavior such as isolation from the group, hyperactivity, apathy, reduced response to handling, irregular, reduced, labored, or increased respiration, small wounds, thinning or ruffled fur, signs of dehydration including sunken eyes, persistent skin folds and reduced skin turgor, dry, inflamed, or watery eyes, bloody stool or diarrhea, sluggish or uncoordinated gait, limping or paralysis, abnormal behavior during handling including increased vocalization suggestive of pain, altered body temperature with the animal feeling cold to the touch, weight loss ≥ 20% over 2 days compared to baseline body weight, and weight gain > 30% or pronounced edema due to fluid retention.

**Figure 1:**
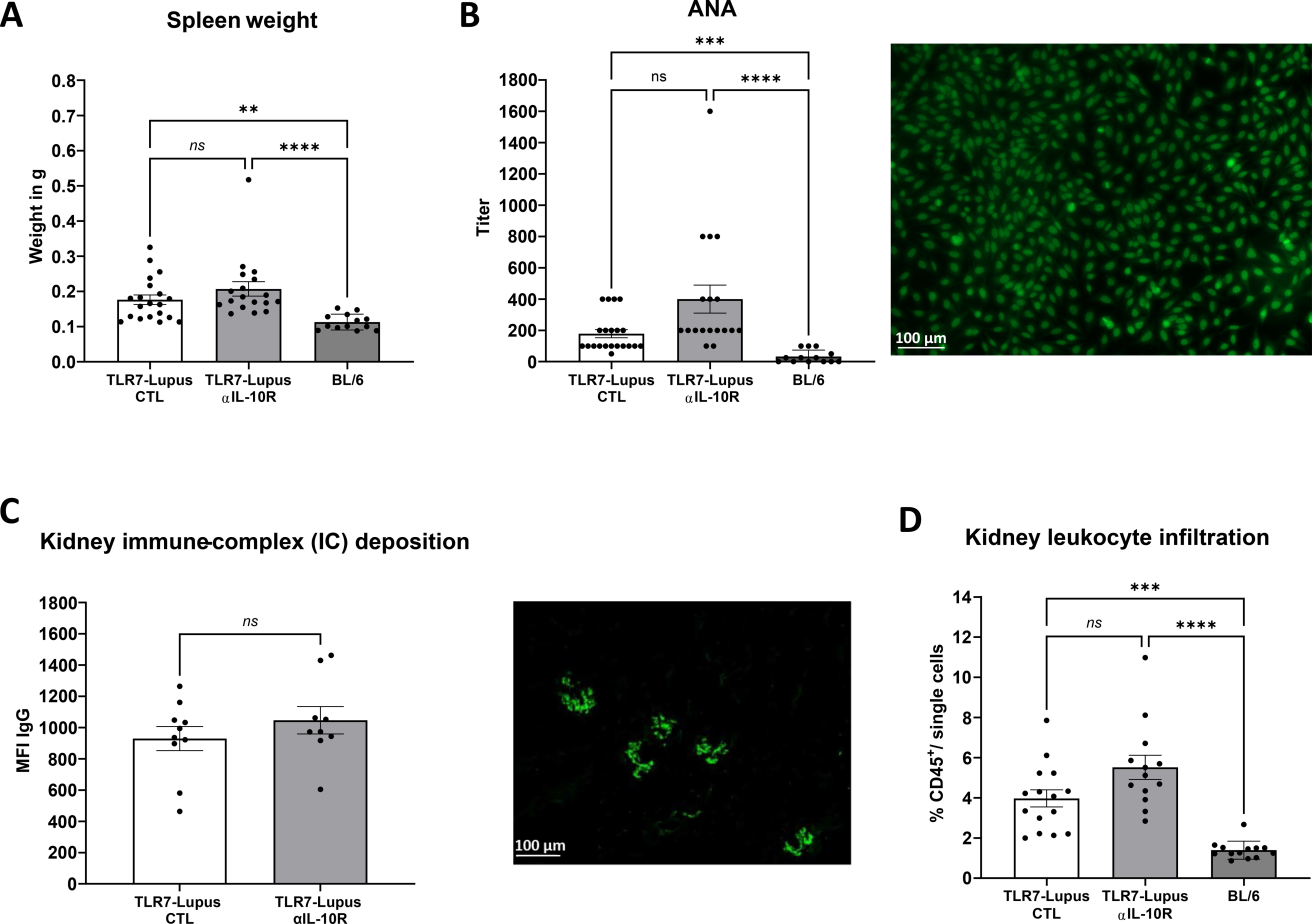
*In vivo* effects of anti-IL-10R application on disease progression in a TLR7-induced mouse model of mild lupus-like disease. C57BL/6 mice were treated epi-cutaneously with Imiquimod for 12 weeks (TLR7-Lupus) or were left untreated (BL/6). During the final 6 weeks of treatment, Imiquimod-treated mice received either an anti-IL-10R antibody or isotype control (*n* (TLR7-Lupus α-IL-10R) = 9–18 mice; *n* (TLR7-Lupus CTL) = 10–20 mice, *n* (BL/6) = 13 mice). Clinical disease progression was assessed by: **(A)** lymphoproliferation as indicated by splenomegaly (spleen weight); **(B)** serum anti-nuclear antibody (ANA) titers; **(C)** renal IgG deposition and **(D)** renal infiltration by CD45^+^ leukocytes. Results are expressed as scatter blots with mean ± SEM; each data point represents an individual mouse. For statistical analysis normality of data distribution was assessed using the Shapiro-Wilk test. **(A, B, D)** Statistical comparisons were calculated with a Kruskal–Wallis test (non-parametric data). **(C)** Statistical comparison was calculated using an unpaired *t*-test. *p* ≤ 0.05 was considered significant, *p* > 0.05 is indicated as *ns*, not significant. * = *p*<0.05, ** = *p*<0.01, *** = *p*<0.001, **** = *p*<0.0001. *ANA, Anti-nuclear-antibodies; g, gramm.*.

**Figure 2:**
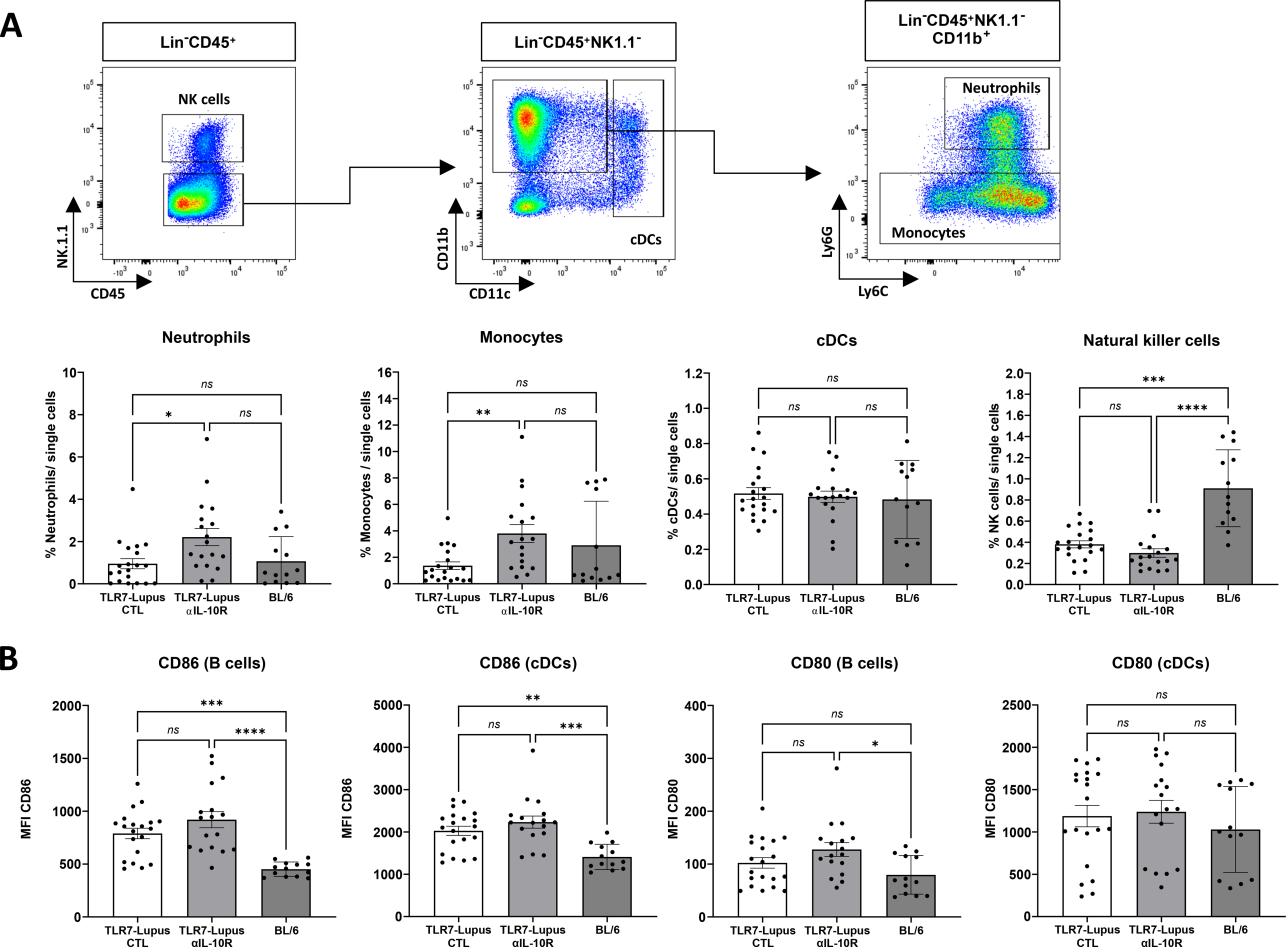
*In vivo* effects of anti-IL-10R application on innate immune cells in a TLR7-induced mouse model of mild lupus-like disease. C57BL/6 mice; were treated epi-cutaneously with Imiquimod for 12 weeks (TLR7-Lupus) or were left untreated (BL/6) mice. During the final 6 weeks of treatment, Imiquimod-treated mice received either an anti-IL-10R antibody or isotype control (*n* (TLR7-Lupus α-IL-10R) = 15-18 mice; *n* (TLR7-Lupus CTL) = 20 mice; *n* (BL/6) = 13 mice). Mice were then sacrificed for evaluation of immunologic changes by flow cytometry. Depicted are gating strategies to define the following immune cell populations and the analysis of their frequencies: **(A)** Lin^-^CD45^+^NK.1.1^-^CD11b^+^CD11c^int/-^Ly6G^+^ neutrophils, Lin^-^CD45^+^NK.1.1^-^CD11b^+^CD11c^int/-^Ly6G^-^ monocytes, Lin^-^CD45^+^NK.1.1^-^CD11c^hi^ cDCs, and Lin^-^CD45^+^NK1.1^+^ natural killer cells. **(B)** Expression of co-stimulatory molecules CD80 and CD86 on B cells and cDCs. Results are expressed as scatter blots with mean ± SEM; each data point represents an individual mouse. For statistical analysis normality of data distribution was assessed using the Shapiro-Wilk test. Statistical comparisons were done either by an ordinary one-way ANOVA (normally distributed data) or a Kruskal–Wallis test (non-parametric data). *p* ≤ 0.05 was considered significant, *p* > 0.05 is indicated as *ns*, not significant. * = *p*<0.05, ** = *p*<0.01, *** = *p*<0.001, ****= *p*<0.0001. *Lin, TCR-β, CD19; cDC, classical dendritic cell*.

**Figure 3:**
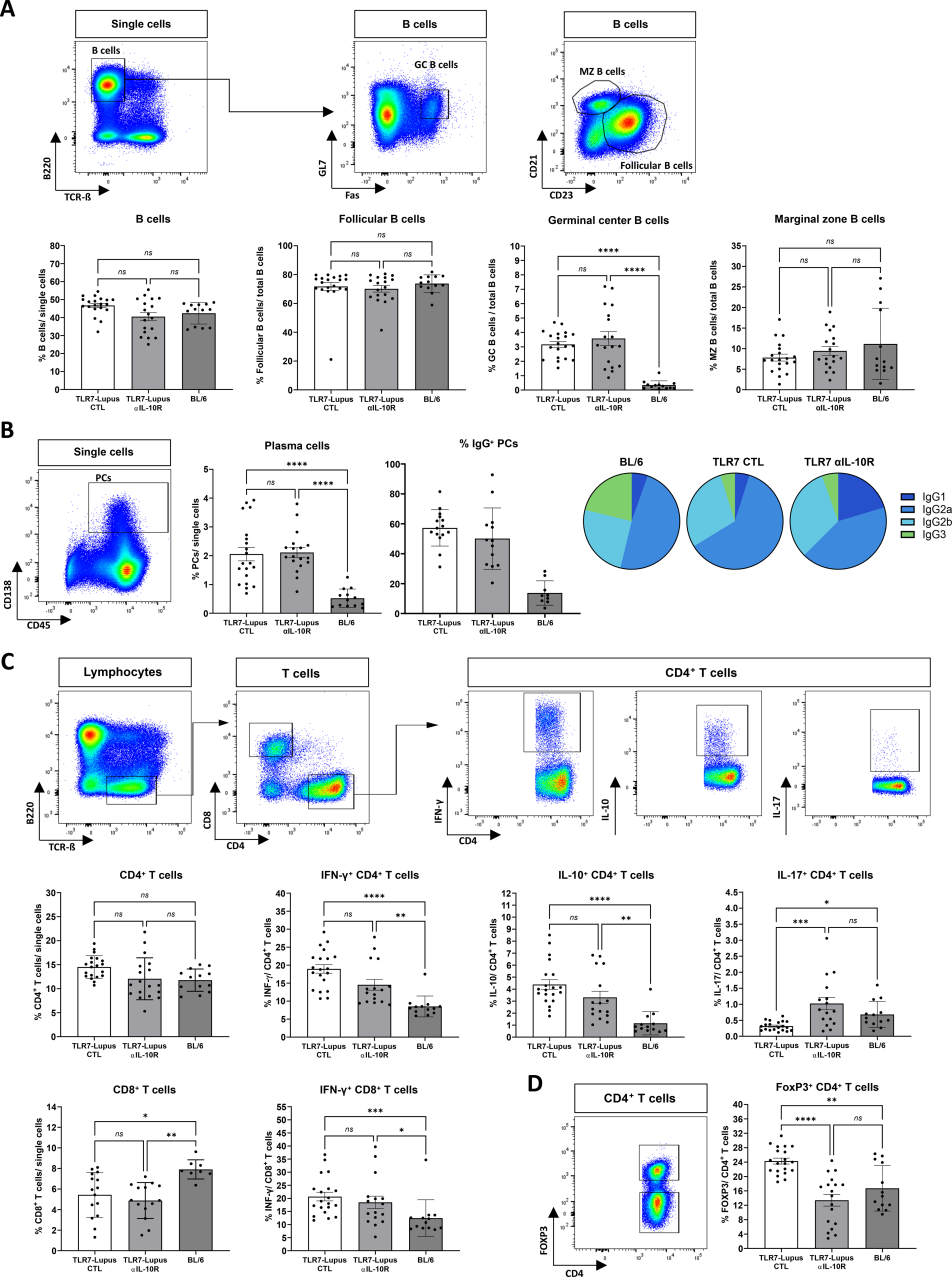
*In vivo* effects of anti-IL-10R application on adaptive immune cells in a TLR7-induced mouse model of mild lupus-like disease. C57BL/6 mice were treated epi-cutaneously with Imiquimod for 12 weeks (TLR7-Lupus) or were left untreated (BL/6). During the final 6 weeks of treatment, Imiquimod-treated mice received either an anti-IL-10R antibody or isotype control (*n* (TLR7-Lupus α-IL-10R) = 13–18 mice; *n* (TLR7-Lupus CTL) = 15–20 mice; *n* (BL/6) = 13 mice). Mice were then sacrificed for evaluation of immunologic changes by flow cytometry. Depicted are gating strategies to define the following immune cell populations and the analysis of their frequencies: **(A)** B220^+^TCR-β^-^ B cells, CD23^hi^CD21^low^ follicular B cells, Fas^hi^GL7^hi^ GC B cells and CD21^hi^CD23^low^ MZ B cells. **(B)** CD138^hi^ PCs and percentage of PCs expressing IgG and distribution of IgG1, IgG2a, IgG2b, and IgG3. **(C)** CD4^+^ and CD8^+^ T cells and cytokine expression (IFN-γ, IL-10, IL-17). **(D)** FoxP3^+^ T_reg_ cells. Results are expressed as scatter blots with mean ± SEM; each data point represents an individual mouse. For statistical analysis normality of data distribution was assessed using the Shapiro-Wilk test. Statistical comparisons were done either by an ordinary one-way ANOVA (normally distributed data) or a Kruskal–Wallis test (non-parametric data). *p* ≤ 0.05 was considered significant, *p* > 0.05 is indicated as *ns*, not significant. * = *p*<0.05, ** = *p*<0.01, *** = *p*<0.001, ****= *p*<0.0001. *GC, germinal center; MZ, marginal zone; plasma cells, PCs; IgG, Immunoglobulin G*.

To investigate the *in vivo* effects of anti-IL-10R on disease progression and immune status in a TLR7-induced mouse model of mild lupus-like disease (**Figures 1**–**3**, **Table 1**), the study was initiated with the following group sizes: n (TLR7-Lupus α-IL-10R) = 18 mice; n (TLR7-Lupus CTL) = 20 mice; and n (BL/6) = 13 mice. In total, the experiment was conducted in four independent cohorts, and the data were pooled for analysis. Not all included mice were considered in every analysis. Reduced sample sizes in individual experiments were due to failed experiments, lost or uncollected samples, and the exclusion of data points identified as outliers in statistical analyses.

**Table 1:** *In vivo* effects of anti-IL-10R application on immune status in a TLR7-induced mouse model of mild lupus-like disease.

Organ	TLR7-Lupus CTL	TLR7-Lupus αIL-10R	C57/BL6J	TLR7-Lupus CTL vs. αIL-10R	TLR7-Lupus CTL vs. C57/BL6J
Spleen	Mean ±SEM	Mean ±SEM	Mean ±SEM	*p-value*	*p-value*
Distribution of adaptive immune cell populations [%]					
CD4^+^ T cells	15 ± 0.52	12 ± 1.0	12 ± 0.64	*0.0582*	*0.0527*
T_reg_	24 ± 0.82	13 ± 1.6	17 ± 1.8	*< 0.0001*	*0.0066*
IFN-γ	19 ± 1.2	15 ± 1.5	8.5 ± 0.80	*0.2101*	*< 0.0001*
IL-10	4.4 ± 0.81	3.3 ± 0.51	1.2 ± 0.27	*0.2759*	*< 0.0001*
IL-17	0.32 ± 0.032	1.0 ± 0.20	0.68 ± 0.11	*0.0004*	*0.0183*
CD8^+^ T cells	5.4 ± 0.56	4.9 ± 0.47	7.9 ± 0.33	*0.6968*	*0.0104*
IFN-γ	21 ± 1.6	18 ± 2.4	12 ± 1.9	*0.7960*	*0.0006*
B cells	47 ± 1.2	40 ± 2.3	42 ± 1.7	*0.1081*	*0.1762*
Follicular B cells	72 ± 2.8	70 ± 2.3	74 ± 1.7	*> 0.9999*	*> 0.9999*
Germinal center B cells	3.2 ± 0.21	3.6 ± 0.48	0.34 ± 0.082	*> 0.9999*	*< 0.0001*
Marginal zone B cells	7.8 ± 0.84	9.4 ± 1.1	11 ± 2.4	*> 0.9999*	*> 0.9999*
Plasma cells	2.1 ± 0.24	2.1 ± 0.17	0.52 ± 0.089	*0.9749*	*< 0.0001*
IgG1	4.4 ± 0.81	6.4 ± 1.5	1.5 ± 0.27	*0.8513*	*0.0157*
IgG2a	30 ± 2.5	27 ± 3.4	6.8 ± 2.3	*> 0.9999*	*0.0002*
IgG2b	13 ± 0.82	14 ± 2.6	3.9 ± 0.48	*0.9963*	*0.0025*
IgG3	8.9 ± 1.7	6.9 ± 1.5	1.6 ± 0.25	*0.7573*	*0.0001*
Co-stimulatory molecule expression on B cells [MFI]					
CD80	102 ± 10	128 ± 13	80 ± 10	*0.6024*	*0.3509*
CD86	789 ± 50	920 ± 76	452 ± 19	*0.2196*	*0.0006*
Distribution of innate immune cell populations [%]					
Neutrophils	0.95 ± 0.24	2.2 ± 0.41	1.1 ± 0.32	*0.0198*	*> 0.9999*
Monocytes	1.4 ± 0.29	3.2 ± 0.62	4.0 ± 1.2	*0.0680*	*0.1280*
cDCs	0.51 ± 0.031	0.51 ± 0.035	0.48 ± 0.061	*0.9997*	*0.9145*
Natural killer cells	0.38 ± 0.034	0.30 ± 0.040	0.91 ± 0.10	*<0.0001*	*0.0008*
Co-stimulatory molecule expression on cDCs [MFI]					
CD80	1187 ± 126	1238 ± 135	1029 ± 141	*>0.9999*	*0.8162*
CD86	2027 ± 106	2232 ± 143	1410 ± 83	*>0.9999*	*0.0019*

Broad immune status evaluation in spleens of C57BL/6 mice treated epi-cutaneously with Imiquimod for 12 weeks (TLR7 lupus) or left untreated (BL/6). During the final 6 weeks of treatment, Imiquimod-treated mice received either an anti-IL-10R antibody or isotype control (*n* (α-IL-10R) = 13-18 mice; *n* (isotype) = 15–20 mice; *n* (BL/6) = 8-13 mice). Results are expressed as mean ± SEM. For statistical analysis SEM was calculated using the descriptive statistics tool from GraphPad Prism. Normality of data distribution was assessed using the Shapiro-Wilk test. Statistical comparisons were done either by an ordinary one-way ANOVA (normally distributed data) or a Kruskal–Wallis test (non-parametric data). PC, plasma cell; cDC, classical dendritic cell; SEM, standard error of mean.

**Figure 4:**
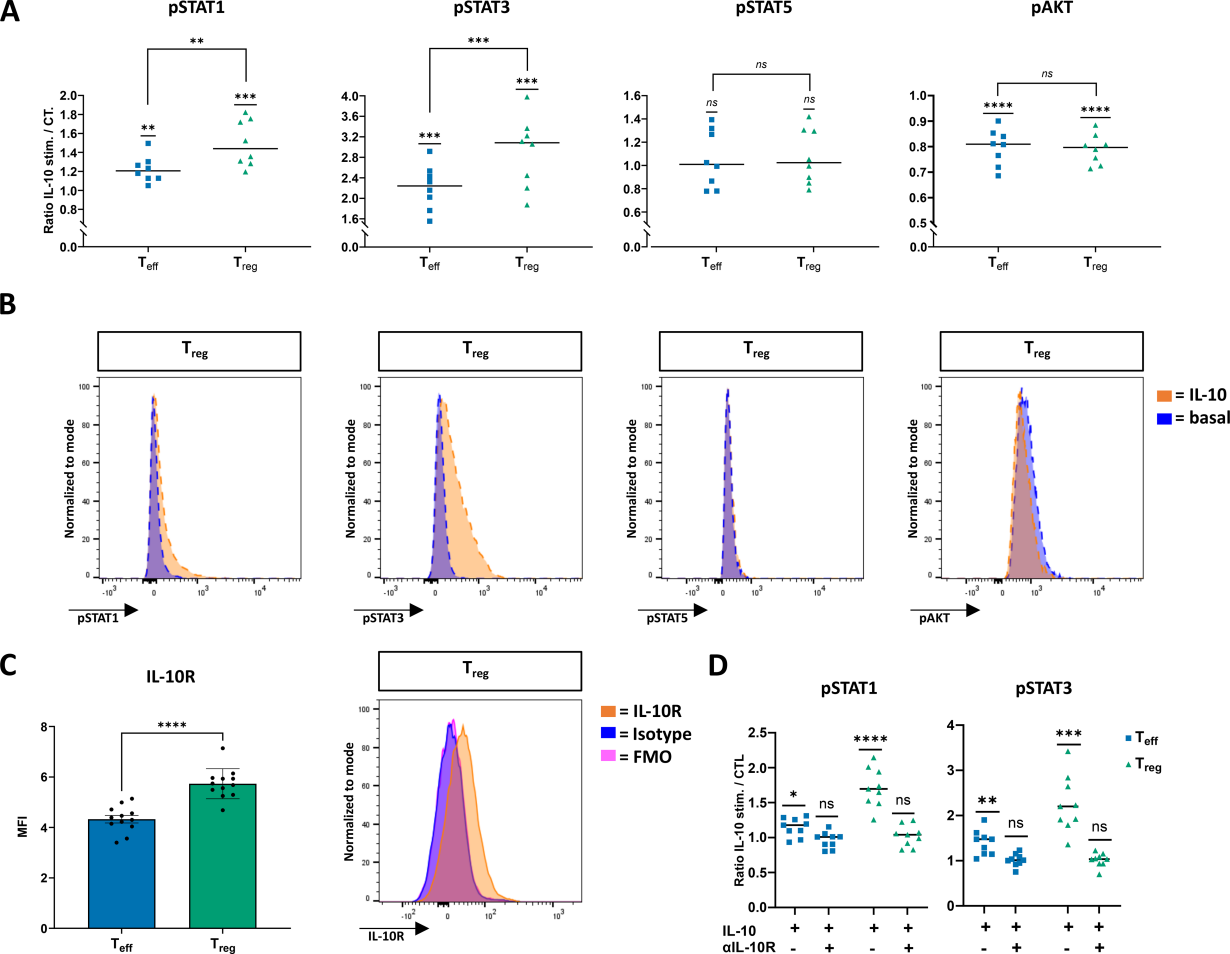
IL-10 activates similar signaling pathways in effector and regulatory T cells in a quantitatively different manner. C57BL/6 mice were treated epi-cutaneously with Imiquimod for 12 weeks (TLR7-lupus) to collect spleen cells for preparation of single-cell suspensions and subsequent flow cytometric analysis. **(A)** Phosphorylation levels (MFI) of STAT1, STAT3, STAT5, and AKT were assessed in T effector (CD4^+^FoxP3^-^ T_eff_) and regulatory T (CD4^+^FoxP3^+^ T_reg_) cells, with and without stimulation with IL-10 for 15 minutes (*n*=8 mice). Results are presented as scatter plots with median; each data point represents an individual mouse. Shown are fold changes in IL-10-stimulated versus unstimulated conditions. The statistical significance of MFI changes upon IL-10 stimulation was determined using a one-sample *t*-test with the hypothetical mean of 1. A paired *t*-test was used for comparison of MFI changes between T_regs_ and T_effs_. **(B)** Additionally shown are representative histograms of MFI changes in T_regs_ with and without IL-10 stimulation. **(C)** Mean fluorescence intensity (MFI) of IL-10R expression was determined on T_regs_ and T_effs_ *ex vivo* (*n*=12 mice). Depicted are scatter plots with mean ± SEM (each data point represents an individual mouse) and representative histograms of IL-10R expression on T_regs_, including isotype and FMO-control. An unpaired *t*-test was used to determine differences in IL-10R expression. **(D)** Phosphoryla.on levels (MFI) of STAT1 and STAT3 were assessed T_effs_ and T_regs_ cells, with and without stimulation with IL-10 for 15 minutes. One group of IL-10-stimulated cells was additionally treated with anti-IL-10R; the antibody was added to the culture 15 minutes prior to IL-10 stimulation (*n*=9 mice). Results are presented as scatter plots with median; each data point represents an individual mouse. Shown are fold changes in IL-10-stimulated as well as IL-10-stimulated and anti-IL-10R-pre-treated versus unstimulated conditions. The statistical significance of MFI changes upon IL-10 stimulation was determined using a one-sample *t*-test with the hypothetical mean of 1. *p* ≤ 0.05 was considered significant, *p* > 0.05 is indicated as *ns*, not significant. * = *p* < 0.05, ** = *p* < 0.01, *** = *p* < 0.001, ****= *p* < 0.0001. *T_reg_, T regulatory cells; T_eff_, T effector cells; pSTAT1/3/5, phosphorylated Signal Transducer and Activator of Transcription 1/3/5; pAKT, phosphorylated Protein Kinase B; MFI, Mean Fluorescence Intensity; stim., stimulated; ctrl., control IL-10R, IL-10 receptor; FMO, fluorescence minus one.*.

The analysis of IL-10-induced signaling pathway activation included 8 mice (**Figure 4A**), while IL-10R expression was assessed in 12 mice (**Figure 4C**). The investigation of the dependence of IL-10 effects on IL-10R-mediated signaling included 9 mice (**Figure 4D**). Each of these experiments was performed in 3 independent cohorts, and the data were subsequently pooled. All included animals were considered as individual data points. Where indicated, experiments were performed in independent cohorts that were processed separately under identical experimental conditions. Prior to pooling, data from each cohort were assessed individually to confirm comparable distributions and consistent trends across cohorts. As no relevant cohort-specific differences were observed, the datasets were subsequently pooled for downstream analyses to increase statistical power.

### 2.2 Assessment of anti-nuclear antibodies

The titers of anti-nuclear antibodies (ANA) were determined by immunofluorescence staining. Mouse serum samples, along with a known positive control (sera of NZB/W F1 mice with established nephritis) and a negative control (PBS), were diluted and applied to designated fields on NovaLite^®^ Hep-2 ANA microscope slides. Slides were incubated at room temperature for 30 minutes, then gently rinsed with PBS to prevent cross-contamination. Following two 5-minute washes in PBS, goat anti–mouse IgG–FITC (Southern Biotech, 1030-02) was applied and incubated for 30 minutes in the dark. After further washing steps, slides were mounted with mounting medium and covered with cover slips. The highest serum dilution showing a positive signal was evaluated independently by two reviewers using a fluorescence microscope, and the mean titer was calculated.

### 2.3 Flow cytometry

For preparation of single-cell suspensions, spleens and kidneys were mechanically dissociated. Red blood cells (RBCs) were lysed in all samples by a 5-minute incubation with RBC lysis buffer. To block non-specific Fc receptor binding, cells were pre-incubated with anti-CD16/32 antibodies (BioLegend, 101330). Surface staining was performed on ice for 25 minutes using either biotin- or fluorochrome-conjugated monoclonal antibodies diluted in 2% FCS/PBS. For intracellular staining, cells were fixed and permeabilized using BD Cytofix/Cytoperm (BD Biosciences). For intranuclear staining, including FoxP3, the FoxP3/Transcription Factor Staining Buffer Set (eBioscience) was used. For cytokine detection, cells were stimulated for 4 hours at 37 °C and 5% CO_2_ with a solution containing 50 ng/ml PMA (Sigma-Aldrich), 1 µg/ml ionomycin (Sigma-Aldrich), and 1 µg/ml Brefeldin A (eBioscience) prior to staining and fixation. Intracellular staining of signaling molecules was performed as follows: for signaling assays, 4 × 10^6^ freshly isolated splenocytes were rested at 37 °C in R0-medium (RPMI 1640, Gibco) for 15min. Phosphorylation of AKT, STAT1, STAT3 and STAT5 was determined after incubation at 37 °C in the presence or absence of IL-10 (100ng/ml, Peprotech, 210-10) for 15min. One group of IL-10-stimulated cells was additionally treated with neutralizing anti-IL-10R (Bio X Cell, clone 1B1.3A, 10μg/ml); the antibody was added to the culture 15 minutes prior to IL-10 stimulation. Unspecific binding or general antibody-related effects were ruled out in preceding test experiments, in which both IL-10–treated and untreated mice were pre-incubated with an isotype control. After fixation at 2-8 °C for 50min with TFP Fix/Perm buffer (BD Pharmigen Transcription Factor Phospho Buffer Set, Cat. No. 565575) and washing with the TFP perm/wash buffer, the cells were permeabilized with ice cold Perm buffer III (both BD Biosciences, La Jolla, CA, USA) on ice for 20min. Additional washing steps were conducted and the cells were then transferred to a 96 well plate (approximately 1 x 10^6^ cells per well) and stained with the respective antibodies for 40–50 min on ice in the dark.

### 2.4 Antibodies used for flow cytometry

Anti-mouse CD103 Brilliant Violett 605 (*BD Biosciences, 748257*), Anti-mouse CD11b FITC (*BioLegend, 101206*), Anti-mouse CD11b PE-Cyanine7 (*Biolegend, 101215*), Anti-mouse CD11c APC (*BioLegend, 117309*), Anti-mouse CD11c PE-Cyanine7 (*BioLegend, 117318*), Anti-mouse CD138 Brilliant Violet 421 (*BioLegend, 142508*), Anti-mouse CD138 Brilliant Violet 605 (*BioLegend, 142531*), Anti-mouse CD161 (NK.1.1) Biotin (*BioLegend, 108704*), Anti-mouse CD161 (NK1.1) APC-Cyanine7 (*BioLegend, 108723*), Anti-mouse CD161 (NK1.1) FITC (*BioLegend, 108705*), Anti-mouse CD19 APC (*BioLegend, 115511*), Anti-mouse CD19 Biotin (*BioLegend, 115504*), Anti-mouse CD19 PE-Cyanine7 (*BioLegend, 115520*), Anti-mouse CD21 FITC (*BD Pharmigen, 553818*), Anti-mouse CD210/IL-10R PE (*BioLegend, 112705*), Anti-mouse CD23 PE (*BD Pharmigen, 553139*), Anti-mouse CD267/TACI APC (*invitrogen, 17-5942-82*), Anti-mouse CD267/TACI PE (*BioLegend, 133404*), Anti-mouse CD275/ICOS-L PE (*BioLegend, 107405*), Anti-mouse CD317/PDCA-1 PE (*eBioscience, 12-3172-82*), Anti-mouse CD4 Brilliant Violett 711 (*BioLegend, 100549*), Anti-mouse CD4 PE-Cyanine7 (*BioLegend, 100421*), Anti-mouse CD44 PE (*BioLegend, 103023*), Anti-mouse CD45 APC-Cyanine7 (*BioLegend, 103116*), Anti-mouse CD45 Brilliant Violett 605 (*BioLegend, 103155*), Anti-mouse CD45 eFlour 506 (*eBioscience, 69-0451-82*), Anti-mouse CD45 FITC (*BioLegend, 103108*), Anti-mouse CD45R/B220 APC-Cyanine7 (*BioLegend, 103224*), Anti-mouse CD45R/B220 Pacific Blue (*BioLegend, 103230*), Anti-mouse CD80 APC-Fire 750 (*BioLegend, 104739*), Anti-mouse CD86 APC (*BioLegend, 105011*), Anti-mouse CD8α PE (*BioLegend, 100707*), Anti-mouse CD8α PerCP (*BioLegend, 100732*), Anti-mouse CD95/Fas PE (*BD Pharmigen, 554258*), Anti-mouse CXCR5 Biotin (*BD Pharmigen, 551960*), Anti-mouse FoxP3 APC (*Invitrogen, 17-5773-82*), Anti-mouse FoxP3 Biotin (*Invitrogen, 13-5773-82*), Anti-mouse FoxP3 Brilliant Violett 421 (*BioLegend, 126419*), Anti-mouse FoxP3 PE (*eBioscience, 12-5773-82*), Anti-mouse GL7 FITC (*BD Pharmigen, 553666*), Anti-mouse IFN-γ APC (*BioLegend, 505809*), Anti-mouse IgA PE (*Invitrogen, 12-5994-81*), Anti-mouse IgG1 FITC (*BD Pharmigen, 553443*), Anti-mouse IgG1 PE-Cyanine7 (*BioLegend, 406613*), Anti-mouse IgG2a FITC (*BD Pharmigen, 553390*), Anti-mouse IgG2b APC (*BioLegend, 406711*), Anti-mouse IgG3 BV421 (*BD Horizon, 565808*), Anti-mouse IgG3 FITC (*BD Pharmigen, 553403*), Anti-mouse IgM PE-Cyanine7 (*BioLegend, 406514*), Anti-mouse IL-10 FITC (*BioLegend, 505006*), Anti-mouse IL-17A PE (*BD Pharmigen, 559502*), Anti-mouse Ki67 APC (*BioLegend, 652405*), Anti-mouse Ly6C PE-Cyanine7 (*BioLegend, 117318*), Anti-mouse Ly6G Biotin (*BioLegend, 127604*), Anti-mouse Ly6G Pacific Blue (*BioLegend, 127611*), Anti-mouse TCR-ß APC-Cyanine7 (*BioLegend, 109220*), Anti-mouse TCR-ß Biotin (*BioLegend, 109204*), Anti-mouse TCR-ß PerCP-Cyanine5.5 (*BioLegend, 109227*), anti-STAT3 Phospho Alexa Flour 647 (*BioLegend, 651008*), mouse anti-STAT1 Alexa Flour 647 (*BD Pharmigen/BD Phosphoflow™, 612597*), mouse anti-STAT5 Alexa Flour 488 (*BD Pharmigen/BD Phosphoflow™, 612598*), Phospho-Akt (Ser473) Rabbit mAb AF488 (*Cell signaling, 4071S*), Streptavidin PerCP-Cyanine5.5 (*eBioscience, 45-4317-82*), Streptavidin APC (*BioLegend, 405207*), Streptavidin PE (*BD Pharmigen 554061*).

Adapted from previous publications (33), the following immune cell subsets were identified: B cells (%B220^+^TCR-ß^-^/single cells), follicular B cells (%CD23^hi^CD21^low^/B cells), marginal zone B cells (%CD21^hi^CD23^low^/B cells), germinal center B cells (%Fas^hi^GL7^hi^/B cells), plasma cells (%CD138^hi^/single cells), CD4^+^ (%CD4^+^CD8^-^B220^-^TCR-ß^+^ or CD4^+^CD8^-^B220^-^NK.1.1^-^/single cells) and CD8^+^ T cells respectively (%CD4^-^CD8^+^B220^-^TCR-ß^+^ or CD4^-^CD8^+^B220^-^NK.1.1^-^/single cells), expression of IL-10, IL-17 or IFN-γ on CD4^+^ T-cells (%IL-10/IL-17/IFN-γ/CD4^+^ T cells), T regulatory cells (T_regs_) (%FoxP3^+^/CD4^+^ T cells), T effector cells (T_eff_) (FoxP3^-^/CD4^+^ T cells), conventional dendritic cells (cDCs) (%CD45^+^TCR-ß^-^CD19^-^NK1.1^-^CD11c^hi^/single cells), natural killer cells (%CD45^+^TCR-ß^-^CD19^-^NK.1.1^+^/single cells), neutrophils (CD45^+^TCR-ß^-^CD19^-^NK1.1^-^CD11b^+^CD11c^int/-^Ly6G^+^/single cells), monocytes (CD45^+^TCR-ß^-^CD19^-^NK1.1^-^CD11b^+^CD11c^int/-^Ly6G^-^/single cells) and expression of CD80 and CD86 on cDCs and B cells. Leukocyte infiltration in the kidney was determined by CD45^+^/single cells.

### 2.5 Immunofluorescent staining

Following organ harvest, portions of the kidney specimens were embedded in O.C.T. compound (Tissue-Tek), snap-frozen on dry ice, and sectioned at 8 µm thickness using a cryotome (Zeiss, Germany). Dried kidney sections were fixed in pre-cooled acetone for 10 minutes on ice, then washed with PBS. For blocking, sections were incubated for 1 hour at room temperature in PBS containing 10% FCS and 1% BSA. Subsequently, sections were stained for 2 hours at room temperature with FITC-conjugated polyclonal goat anti–mouse IgG (Southern Biotech). After three washes in PBS, nuclei were counterstained with DAPI (Roche) for 10 minutes at room temperature. Finally, sections were mounted using fluorescent mounting medium (Dako) and imaged with a fluorescence microscope (AxioImager 2, Zeiss) using a monochrome camera at 20× magnification. For quantification of IgG deposition, the mean fluorescence intensity was measured in 30 glomeruli per section.

### 2.6 Statistical analysis

For statistical analysis, GraphPad Prism version 10.4.2 for Windows (GraphPad Software, Boston, Massachusetts USA, www.graphpad.com) was used. For statistical analysis, normality and lognormality were first assessed using the Shapiro–Wilk test. A statistical comparison between two experimental groups was performed using a *t*-test (normally distributed data), or a Mann–Whitney U-test (non-parametric data). A statistical comparison between three experimental groups was performed using an ordinary one-way ANOVA (normally distributed data) or a Kruskal–Wallis test (non-parametric data). A one sample *t*-test with a hypothetical mean of 1 was used to determine fold-changes in phosphorylation levels of different signaling molecules with and without stimulation. Generally, outliers were determined *via* the Robust regression and outlier removal (ROUT) method. Generally, *p* ≤ 0.05 was considered significant. In all figures *ns* (not significant) describes a *p* value >0.05, * = *p* < 0.05, ** = *p* < 0.01, *** = *p* < 0.001, ****= *p* < 0.0001.

## 3 Results

### 3.1 Epi-cutaneous treatment with the TLR7 agonist Imiquimod induces mild lupus-like disease in C57BL/6 mice that is further triggered by the administration of anti-IL-10R

Toll-like receptor 7 (TLR7) is not only a key driver of lupus but also closely linked to IL-10, playing a crucial role in balancing pro-inflammatory and anti-inflammatory, regulatory responses (34, 35). For instance, TLR7 promotes germinal center reactions that trigger autoimmunity (36), as well as autoantibody production and lupus-associated monocytosis in NZB/W F1 mice (37). Conversely, repeated stimulation of TLR7 with synthetic agonists can induce tolerance and suppress inflammation in autoimmune and tumor models, partly by upregulating negative regulators of TLR signaling and immunosuppressive cytokines like IL-10 and transforming growth factor β1 (TGF-β1) (38, 39). Moreover, the TLR7 agonist imiquimod (IMQ) is used in an inducible mouse model for SLE and was also employed in our study (40).

Although this TLR7-induced lupus model, driven largely by plasmacytoid dendritic cells and type I interferon signaling relevant to human disease, is increasingly used to study lupus pathogenesis, systematic analyses of immune phenotypes and nephritis development in the C57BL/6 strain remain limited. We therefore addressed this gap by comparing TLR7-treated mice with untreated C57BL/6 controls in addition to assessing the impact of IL-10R antagonism. To induce lupus-like disease, female C57BL/6 mice received topical IMQ three times weekly for 12 weeks. To evaluate the effects of IL-10 on disease progression and immune changes, C57BL/6 mice treated with IMQ (as described above) received anti-IL-10R antibodies or isotype control during the last 6 weeks.

Compared to age-matched untreated controls, IMQ-treated mice developed marked splenomegaly indicative of lymphoproliferation (**Figure 1A**) and detectable but low-titer ANAs (**Figure 1B**). Notably, ANAs were also sporadically detectable in untreated control mice, which is consistent with reports showing that most mice of the C57BL/6 strain spontaneously produce IgG ANAs at 8–12 months (41) (**Figure 1B**). Unlike IMQ-treated FVB/N and BALB/c mice (40), no significant nephritis was observed based on edema and proteinuria assessments (data not shown). However, histological analysis revealed mild IC deposition in the glomeruli (**Figure 1C**) and significantly increased leukocyte infiltration in the kidneys (**Figure 1D**) compared to untreated controls.

While anti-IL-10R treatment did not affect lymphoproliferation (**Figure 1A**) or IC deposition in the kidneys (**Figure 1C**), a moderate, but not-significant increase of ANA titers (**Figure 1B**) and kidney-infiltrating leukocytes was induced by IL-10R blockade (**Figure 1D**).

In summary, IMQ treatment in C57BL/6 mice induces a mild lupus-like phenotype without overt nephritis, making it suitable for studying lymphoproliferation and associated immunological changes, but limited for assessing overt nephritis as a primary readout. Using this model, we find that IL-10R blockade further promotes disease features.

### 3.2 Epi-cutaneous TLR7 stimulation and IL-10R blockade induce immune remodeling and elicit pro- and anti-inflammatory immune responses

We also investigated immunologic changes accompanying the mild lupus-like phenotype and how IL-10R blockade reshapes innate and adaptive immune responses in the spleen.

Within the innate immune compartment, relative frequencies of monocytes, conventional dendritic cells (cDCs), and neutrophils remained largely unchanged in un- versus IMQ-treated mice (**Figure 2A**). IMQ-treated mice showed a significant relative decrease in NK cells compared to age-matched C57BL/6 controls (**Figure 2A**). Regarding co-stimulatory molecules, CD86 expression was elevated on B cells and cDCs of IMQ-treated compared to untreated C57BL/6 control mice, while CD80 levels showed no significant difference (**Figure 2B**).

Treatment with anti-IL-10R in IMQ-treated mice resulted in a significant relative increase in monocytes and neutrophils (**Figure 2A**) compared with IMQ-treated mice receiving isotype control. In contrast, anti-IL-10R treatment did not affect the frequencies of cDCs or NK cells, nor did it change expression levels of CD80 and CD86 (**Figures 2A, B**).

Within the adaptive immune compartment, IMQ- compared to untreated mice showed similar proportions of total B cells, but a significant increase in germinal center (GC) B cell and plasma cell (PC) differentiation, with elevated expression of all IgG subclasses (IgG1, IgG2a, IgG2b, and IgG3) in plasma cells (**Table 1**, **Figures 3A, B**). The overall frequencies of B cells were largely comparable to those in controls, whereas CD8^+^ T cells showed a modest but significant reduction in lupus-like disease, while CD4^+^ T cells were slightly increased (**Figures 3A, C**). Affecting both CD4^+^ and CD8^+^ T cells, TLR7 agonist treatment altered their differentiation: IFN-γ expression increased, while IL-17 expression on CD4^+^ T cells decreased (**Figure 3C**), despite generally low baseline levels. Additionally, IMQ-treated mice exhibited higher IL-10 expression and increased frequencies of FoxP3^+^ T_regs_ (**Figures 3C, D**) compared to untreated controls.

The treatment with anti-IL-10R in IMQ-treated mice did not alter B cell differentiation, IgG subclass expression in PCs, frequencies of cDCs and NK cells, or the expression of co-stimulatory molecules compared with IMQ-treated mice receiving isotype control (**Figures 2**, **3**). In the T cell compartment, anti-IL-10R treatment led to a marked reduction in T_regs_ and a trend toward fewer IL-10–producing CD4^+^ T cells. It also led to a slight, although non-significant decrease in IFN-γ expression in both CD4^+^ and CD8^+^ T cells, while significantly increasing IL-17 expression in CD4^+^ T cells (**Figures 3C, D**) compared with IMQ-treated mice receiving isotype control.

Altogether, IMQ treatment in C57BL/6 mice and IL-10R antagonism induce a complex pattern of both pro- (increased IL-10 expression by T cells and elevated T_reg_ frequencies) and anti-inflammatory immune changes (increased IL-17 and IFN-γ expression by T cells, enhanced PC differentiation of B cells including expression of IgG isotype, increased expression of co-stimulatory molecules by B cells and myeloid cells and increased frequencies of neutrophils and monocytes).

### 3.3 IL-10 activates similar signaling pathways in effector and regulatory T cells in a quantitatively different manner

Our results demonstrate that in TLR7-induced lupus, IL-10 exerts both pro- and anti-inflammatory effects. Among the analyzed cell subsets, this dual role was most evident in CD4^+^ T cells, where IL-10 supported the expansion of anti-inflammatory T_regs_ and IL-10^+^ CD4^+^ T cells, but slightly also of pro-inflammatory IFN-γ^+^ CD4^+^ T cells, while decreasing pro-inflammatory IL-17^+^ CD4^+^ T cells (**Figures 3C, D**). The integration of complex signaling pathways and quantitative differences in receptor expression may explain how IL-10 elicits divergent or even opposing effects, allowing for context-dependent functional specialization. Given its pronounced impact on T_reg_ frequencies, we investigated whether IL-10 triggers distinct qualitative and quantitative downstream signaling patterns in CD4^+^FoxP3^+^ T_regs_ versus CD4^+^FoxP3^-^ T_effs_, potentially enabling more selective immune modulation. To address this, we isolated splenocytes from C57BL/6 mice treated topically with IMQ for 12 weeks, as mentioned earlier. Cells were then short-term stimulated *in vitro* with IL-10 to assess activation of STAT1, STAT3, STAT5, and PI3K/Akt signaling pathways, as determined by flow cytometric analysis of phosphorylated STAT1/3/5 and AKT. In both CD4^+^FoxP3^+^ T_regs_ and CD4^+^FoxP3⁻ T_effs_, IL-10 increased pSTAT3 and pSTAT1 levels, while pAKT levels were reduced. IL-10 had no effect on pSTAT5 levels in either population (**Figure 4**). When comparing fold-changes in phosphorylation upon IL-10 treatment, STAT3 signaling was more strongly activated than STAT1 or PI3K/AKT pathways (**Figure 4A**). Notably, both STAT1 and STAT3 activation were more pronounced in CD4^+^FoxP3^+^ T_regs_ than in CD4^+^FoxP3^-^ T_effs_.

Finally, we considered whether the observed differences between CD4^+^FoxP3^+^ T_regs_ and CD4^+^FoxP3^-^ T_effs_ might reflect greater IL-10 responsiveness in CD4^+^FoxP3^+^ T_regs_ due to higher IL-10R expression. Although overall IL-10R levels were very low, CD4^+^FoxP3^+^ T_regs_ displayed significantly higher IL-10R expression compared to CD4^+^FoxP3^-^ T_effs_ (**Figure 4C**). The notion that differences in IL-10 effects between CD4^+^FoxP3^-^ T_eff_ and CD4^+^FoxP3^+^ T_reg_ cells may be explained by differential IL-10R expression levels was further supported by the observation that treatment with a neutralizing anti-IL-10R antibody restored IL-10-induced changes of pSTAT1 and pSTAT3 in both cell populations to baseline levels (**Figure 4D**).

Altogether, we find that IL-10 activates similar signaling pathways in effector and regulatory T cells, most prominent STAT3. We speculate that increased IL-10R expression may be one possible explanation for the increased responsiveness of CD4^+^FoxP3^+^ T_reg_ towards IL-10 treatment and why CD4^+^FoxP3^+^ T_reg_ frequencies were most prominently affected by *in vivo* anti-IL-10R-treatment.

## 4 Discussion

This study examined the role of IL-10 in modulating immune responses and disease pathology in a TLR7-induced model of lupus. While we confirm that IL-10 can exhibit both pro- and anti-inflammatory effects in this mild lupus-like disease model, consistent with findings from other studies, inhibition of IL-10R suggests, that under the conditions employed here, IL-10 overall exerts a modest disease-dampening effect, likely indicating a predominantly regulatory influence on disease progression. The strongest effects were observed in the adaptive immune compartment, where IL-10R blockade led to a marked reduction in CD4^+^FoxP3^+^ T_reg_ frequencies and increased IL-17 expression. Additionally, we sought to identify potential determinants of its stimulatory versus suppressive effects. Although IL-10 activated similar downstream signaling pathways in both effector and regulatory T cells, with STAT3 showing the strongest quantitative activation, suggesting it may serve as the central mediator, we speculate that the slightly higher IL-10R expression on CD4^+^FoxP3^+^ T_regs_ may account for their heightened sensitivity to IL-10 and likely explain their pronounced response to IL-10R blockade compared to T_eff_ *in vivo*.

Whether IL-10 promotes or restrains disease progression in lupus remains a matter of ongoing debate, with divergent outcomes reported in both human and murine studies. Consistent with a previous study from our group (21), multiple reports suggest that the timing of intervention, as well as the specific disease model, clinical manifestation, stage, and activity, critically influence the effects of IL-10 neutralization. Our immunophenotypic analysis confirms that IL-10 can exert both pro- and anti-inflammatory effects. Under the conditions employed here, the mild acceleration of disease progression following *in vivo* IL-10R blockade in the IMQ-induced lupus model suggests that, despite IL-10’s dual roles, its protective effects predominate. In both the current and our earlier study, anti-IL-10R treatment was initiated at a more advanced disease stage, characterized by established immune dysregulation or early nephritis (21), more closely mimicking therapeutic intervention in clinical settings. Supporting this notion, adoptive transfer of *in vitro*-generated polyclonal IL-10–producing CD4^+^ T cells into severely ill NZB/W F1 mice improved lupus pathology (42). Conversely, IL-10 neutralization in NZB/W F1 mice from birth to 8–10 months of age delayed disease onset, as shown by improved survival, and delayed development of proteinuria, glomerulonephritis, and autoantibodies (43). In another lupus model, MRL-Faslpr mice, IL-10 deficiency exacerbated disease severity and autoantibody production (44). Similar protective effects of IL-10 were also reported in B6.TC lupus mice (45).

Disease model, stage, and activity likely shape the pro-inflammatory microenvironment and thereby influence the overall impact of IL-10 on immune responses. This is further underscored by our observation that, although anti-IL-10R treatment, applied at a relatively advanced rather than pre-stage of disease in both models, modestly aggravated lupus progression in both NZB/W F1 mice (21) and the TLR7-induced lupus (this study), the associated immunologic changes differed partially between the models. In both, anti-IL-10R treatment led to a significant increase in neutrophils and monocytes, indicating a possibly conserved effect on the innate immune compartment. However, only in NZB/W F1 mice did IL-10R blockade significantly alter the B cell compartment and promote plasma cell differentiation, suggesting that the impact of IL-10 on humoral immunity was model- and context-dependent. Notably, a dual role of IL-10 in CD4^+^ T cell regulation emerged in both models: neutralization of IL-10 consistently reduced IL-10–producing CD4^+^ T cells and FoxP3^+^ T_regs_, while simultaneously slightly lowering IFN-γ^+^ but increasing IL-17^+^ T cell frequencies. These findings highlight possible pleiotropic effects of IL-10, particularly within the CD4^+^ T cell pool, where it appears to be able to regulate both suppressive and effector functions in a context-sensitive manner. Overall, it remains challenging to attribute the effects of IL-10 to a single immune cell as determining factor. Likewise, it remains unclear whether shared or divergent downstream signaling pathways are engaged. Based on the clinical course observed, we conclude that anti-inflammatory functions of IL-10 may predominate under the conditions employed here, evidenced by suppression of pro-inflammatory innate immune cells such as neutrophils and monocytes, inhibition of T helper 17 (Th17) responses, and expansion of regulatory T cells. These anti-inflammatory actions are consistent with previous reports (5, 46) and were further explored in our study, with particular focus on the adaptive immune compartment. Generally, Foxp3^+^ regulatory T cells (T_regs_) play a crucial role in maintaining immune homeostasis. While most T_regs_ develop in the thymus (natural T_regs_, or nT_regs_), induced T_regs_ (iT_regs_) can also arise from naïve CD4^+^ T cells in the periphery under specific conditions (47). Both populations are modulated by IL-10, which influences not only their frequency but also their suppressive function. For example, in murine colitis models, IL-10 has been shown to stabilize FoxP3 expression in established nT_regs_ (48). Another study demonstrated that IL-10 can signal via STAT3 in T_regs_ to enhance their own IL-10 production, an essential mechanism for controlling Th17-mediated intestinal inflammation (49). Moreover, in combination with TGF-β, IL-10 promotes the expansion of Foxp3^+^ iT_regs_ with increased CTLA-4 expression and enhanced suppressive function (50). The potentiating effect of IL-10 in this study is likely mediated not only through STAT3 signaling but also via inhibition of AKT phosphorylation, which preserves Foxo1 activity—a key transcription factor for T_reg_ differentiation and function. This is particularly relevant to our findings as we observed robust STAT3 activation in both CD4^+^FoxP3^+^ T_regs_ and CD4^+^FoxP3^-^ T_effs_, with a more pronounced effect in CD4^+^FoxP3^+^ T_regs_. In both subsets, we additionally detected reduced AKT phosphorylation. These results align with previous studies showing that IL-10 can inhibit PI3K/AKT signaling (51, 52), contributing to T cell suppression and regulatory immune modulation.

Furthermore, we propose that STAT3 signaling may have contributed to the altered Th_17_/T_reg_ balance observed in anti-IL-10R–treated mice, as the differentiation of these subsets is shaped by the opposing influences of IL-10 and IL-6. Although both cytokines activate STAT3, they trigger contrasting immunological programs: IL-10 promotes anti-inflammatory responses and supports T_reg_ stability and function, while IL-6 fosters pro-inflammatory signaling, critically driving Th17 cell differentiation (53) and simultaneously inhibiting iT_reg_ development (54). One explanation for this functional divergence lies in the differential susceptibility of their downstream signaling to negative regulation by SOCS3. Specifically, IL-6–induced STAT3 activation is highly sensitive to SOCS3-mediated inhibition, whereas IL-10–driven STAT3 signaling is comparatively resistant (55). This distinction likely enables IL-10 to sustain anti-inflammatory responses even in pro-inflammatory environments where IL-6 is present, whereas in the absence of IL-10 signaling, as in anti-IL-10R–treated mice, IL-6–driven pro-inflammatory pathways may dominate, contributing to Th_17_ skewing and impaired T_reg_ homeostasis.

We also investigated whether activation of signaling pathways beyond STAT3 and PI3K/AKT contributed to the observed effects. Under the experimental conditions of our study, we did not detect activation of STAT5 signaling. However, we observed increased phosphorylation of STAT1 in IL-10–treated CD4^+^ T cells, with a stronger signal in CD4^+^FoxP3^+^ T_regs_ compared to CD4^+^FoxP3^-^ T_effs_. This finding is noteworthy, as STAT1, alongside STAT3, is also implicated in IL-27–driven IL-10 production (56). Moreover, by inducing Tbx21 (T-bet) expression, STAT1 functions as a key regulator of IFN-γ–producing T helper cell 1 (Th1) cells (57, 58). This aligns with our observation that anti-IL-10R treatment reduced both IFN-γ, if only slightly, and IL-10 expression in CD4^+^ T cells, suggesting that IL-10 may support the generation or maintenance of IL-10^+^ and IFN-γ^+^ Th1-like cells through STAT1 and/or STAT3 signaling.

IL-10 can also have stimulatory effects, partly mediated by STAT3, enhancing the expression of both IFN-γ and IL-10 itself (49, 59). In autoimmune settings, the generation of Th1 cells expressing IFN-γ and IL-10 likely serves an important role in self-regulating Th1 effector functions, helping to limit excessive inflammation and prevent autoimmunity (60). Additionally, STAT1 signaling has been shown to inhibit the differentiation of both T_regs_ and Th_17_ cells (61, 62). Conversely, STAT1 also plays a role in modulating specialized T_reg_ functions; for instance, T_regs_ deficient in STAT1 fail to control allograft rejection *in vivo* (63).

A limitation of our study is that a portion of the findings is correlative and, conclusions regarding the potential functional effects of IL-10 are based primarily on descriptive data. In addition, parts of the experiments, particularly those addressing signaling, are based on *in vitro* data, and the effects of IL-10 were assessed indirectly using a neutralizing antibody rather than through *in vivo* overexpression. Nevertheless, these findings suggest how signaling mechanisms may account for the divergent or seemingly contradictory functions induced within the same immune cell or by a single cytokine. Immune cells can integrate complex signals through combinatorial cytokine interactions, pathway crosstalk, and quantitative differences in receptor and phosphatase expression, enabling context-dependent functional specialization. In the case of IL-10, our study and others suggest that STAT3 activation may be the primary signaling pathway engaged in CD4^+^ T cell populations. However, under specific conditions or in distinct cell populations, additional pathways such as STAT1, STAT5, or PI3K/Akt/mTORC1 may also be engaged, resulting in diverse and sometimes opposing functional outcomes. The precise downstream signaling events that mediate IL-10’s stimulatory versus suppressive effects, and how these are modulated by the pro-inflammatory milieu in active lupus or other diseases, remain to be fully elucidated. Nonetheless, this complexity may highlight a potential therapeutic avenue of targeted manipulation of signaling pathways. A deeper understanding of IL-10’s pleiotropic nature, combined with refined modulation of its signaling networks, could enable clinical interventions that maximize its beneficial effects while minimizing potential adverse outcomes. Consequently, further research is essential to unravel IL-10’s dual roles and to develop approaches that exploit context-dependent signaling, combinatorial cytokine interactions, and receptor expression dynamics for optimal therapeutic benefit.

## Data Availability

The original contributions presented in the study are included in the article/supplementary material. Further inquiries can be directed to the corresponding author.
